# Complete Genome Sequence of Pseudomonas aeruginosa XN-1, Isolated from the Sputum of a Severe Pneumonia Patient

**DOI:** 10.1128/MRA.00653-20

**Published:** 2020-09-03

**Authors:** Chen Gao, Ying Wang, Yi Zhang, Jinning Wei, Xin Cheng, Jin Zhang, Quanming Zou, Jiang Gu

**Affiliations:** a National Engineering Research Center of Immunological Products, Department of Microbiology and Biochemical Pharmacy, College of Pharmacy, Third Military Medical University, Chongqing, People’s Republic of China; b Department of Respiratory, Hunan Children's Hospital, Hunan, People’s Republic of China; University of Arizona

## Abstract

Pseudomonas aeruginosa is an important opportunistic pathogen with strong virulence and an invasive nature. Here, we report the complete genome of strain XN-1, which was isolated from the sputum of a severe pneumonia patient. The complete genome consists of one chromosome with 6,340,573 bp. Genome annotation predicts 5,974 coding sequences, 64 tRNAs, and 12 rRNAs.

## ANNOUNCEMENT

Pseudomonas aeruginosa, a versatile Gram-negative pathogen, is a major health challenge that causes recalcitrant multidrug-resistant infections, especially in immunocompromised and hospitalized patients (1). Furthermore, it is an opportunistic pathogen responsible for ventilator-acquired pneumonia (VAP). VAP due to P. aeruginosa is usually multidrug resistant and associated with severe infections and increased mortality rates (2). P. aeruginosa is one of the six superbugs that are threats all over the world because of their capacity to become increasingly resistant to all available antibiotics (3, 4). Moreover, the high rate of mutation allows it to evolve rapidly and to adapt to a multitude of conditions (5).

Here, we report the complete genome sequence of strain XN-1, which was isolated from the sputum of a severe pneumonia patient at Southwest Hospital in Chongqing, China. P. aeruginosa XN-1 (CCTCC M2015730) was isolated from a severe pneumonia patient and deposited in the China Center for Type Culture Collection (CCTCC). According to our previous studies (6–8), strain XN-1 has strong virulence. In a mouse model of acute pneumonia, no mouse survived 36 h after challenge with a lethal dose of bacteria (1.0 × 10^7^ CFU) (6, 7). Moreover, XN-1 is resistant to different antibiotics, including carbapenems, imipenem, and ciprofloxacin (6–8). To better understand the virulent nature and resistance mechanisms of XN-1, the genome was sequenced.

P. aeruginosa XN-1, which had been stored at −80°C, was grown on an LB agar plate at 37°C overnight. A single colony was then picked and grown in LB medium at 37°C for 6 h. The cells were collected by centrifugation, and genomic DNA of P. aeruginosa XN-1 was isolated using the DNeasy blood and tissue kit (Qiagen) according to the manufacturer’s protocol. A standard library of 20-kb fragments was established with SMRTbell methods. The genome of the strain was sequenced by single-molecule real-time (SMRT) sequencing using the Pacific Biosciences (PacBio) RS II platform to obtain high-quality data on the original DNA sequence (9). The total number of reads sequenced is 107,702, and the *N*_50_ value is 13,257 bp.

The continuous long reads were assembled using the HGAP v3.0 protocol (10). Polishing and error correction were performed with Quiver v0.9.2 (10). The software GLIMMER (Gene Locator and Interpolated Markov ModelER) v3.02 (11), tRNAscan-SE v1.23 (12), and RNAmmer v1.2 (13) were used to predict gene structure, tRNAs, and rRNAs, respectively. The final assemblies generated by the approach consist of a single circular chromosome of 6,340,573 bp with a mean G+C content of 66.53%. The total genes predicted for XN-1 were 5,974 coding sequences, 64 tRNAs, and 12 rRNAs, including 4 copies each of 5S, 16S, and 23S rRNAs.

A total of 190 tandem repeats and 16 simple sequence repeats were predicted using Tandem Repeats Finder (TRF) v4.09 (14) and MISA v1.0 (15), respectively. The CRISPR structure of the bacterial genome was predicted using MinCED v0.2.0 software (16), and a total of 10 possible CRISPR components were identified. In addition, functional annotation of the genome was curated and enriched using various databases, including the Clusters of Orthologous Groups of proteins (COG) (17), Gene Ontology (GO) (18), Kyoto Encyclopedia of Genes and Genomes (KEGG) (19, 20), Swiss-Prot (21), and NCBI nonredundant protein databases. A summary of the functional annotation of the genome is presented in Table 1. Default parameters were used for all software unless otherwise specified.

**TABLE 1:** Statistical results of functional genome annotation

Database used	No. of genes annotated	% of total annotated
KEGG	2,987	50.00
InterPro	4,995	83.61
Swiss-Prot	4,312	72.18
COG	5,592	93.61
GO	3,436	57.52
NCBI nonredundant protein	4,003	67.01
Total	5,618	94.04

The genome sequence presented here will be a valuable resource for better understanding the pathogenomic evolution of P. aeruginosa XN-1 by comparing this pathogenic isolate to the extant genotypes. The complete genome sequence of P. aeruginosa XN-1 should provide further insights into the pathogenic mechanism and could have implications for defense against this pathogen.

### Data availability.

The whole-genome project for P. aeruginosa strain XN-1 was deposited in the SRA. The BioProject accession number is PRJNA636895, and the BioSample number is SAMN15088354 (SRX8457197).
